# Self-Assembled Array of Tethered Manganese Oxide Nanoparticles for the Next Generation of Energy Storage

**DOI:** 10.1038/srep44191

**Published:** 2017-03-13

**Authors:** Tyler E. Stevens, Charles J. Pearce, Caleah N. Whitten, Richard P. Grant, Todd C. Monson

**Affiliations:** 1 Sandia National Laboratories, Albuquerque, New Mexico 87185, United States

## Abstract

Many challenges must be overcome in order to create reliable electrochemical energy storage devices with not only high energy but also high power densities. Gaps exist in both battery and supercapacitor technologies, with neither one satisfying the need for both large power and energy densities in a single device. To begin addressing these challenges (and others), we report a process to create a self-assembled array of electrochemically active nanoparticles bound directly to a current collector using extremely short (2 nm or less) conductive tethers. The tethered array of nanoparticles, MnO in this case, bound directly to a gold current collector via short conducting linkages eliminates the need for fillers, resulting in a material which achieves 99.9% active material by mass (excluding the current collector). This strategy is expected to be both scalable as well as effective for alternative tethers and metal oxide nanoparticles.

Electrochemical energy storage devices are the leading means of storing charge where size and weight are critical^1^,^2^,^3^,^4^,^5^,^6^. Although both batteries and supercapacitors have seen significant improvements in recent years, end users of these devices still require substantial improvements in energy density, power density, and reliability^2^,^4^,^5^. One of the largest limitations in the design of electrodes for existing batteries and electrochemical capacitors is the use of electrochemically inactive material, such as conductive carbon and binders, which limit the active electrode mass to roughly 70–80%^1^,^7^,^8^,^9^. Additional challenges come from electrode degradation as a function of cycle life, poor charge transfer, the inability to fully utilize multiple oxidation states, and the use of electrolytes with a low thermodynamic voltage window (this is the case primarily with supercapacitors that utilize aqueous electrolytes)^2^,^3^,^6^,^10^,^11^,^12^,^13^. In the case of a high percentage of inactive material, as many devices are currently designed, the conductive carbon is necessary for transporting charge to and from poorly conductive active materials and the binder is necessary to hold the electrode structure together^2^,^4^,^7^,^8^,^14^,^15^. Reliability is also a key metric as devices capable of surviving >1 M cycles would be extremely beneficial^3^,^16^. Another factor that must be considered, yet is sometimes neglected by us as a research community, is the cost of the active electrochemical material relative to the intended application.

While the work presented herein does not address all of the aforementioned issues, it does provide one significant step towards achieving high reliability, energy density, and power density in a single affordable device. Presented here is the development of a synthetic route for manganese oxide (MnO_x_) nanoparticles tethered to a current collector using conductive organic linkers of 2 nm or less. Depending on the specific choice of active MnO_x_ material, this self-assembled structure can be used as an anode for Li-ion batteries (MnO), a cathode for Li-ion and Li metal batteries (MnO_2_), an electrode for an oxide supercapacitor (MnO_2_), or a catalyst for oxygen reduction in Li-air batteries (MnO_2_)^15^,^17^,^18^,^19^,^20^,^21^,^22^,^23^,^24^,^25^,^26^,^27^,^28^,^29^,^30^,^31^,^32^,^33^,^34^,^35^,^36^,^37^,^38^,^39^. The many applications of MnO_x_ gives the synthetic route and nanoparticle structure reported on here a wide breadth of potential applications. In the oxide supercapacitor application, MnO_2_ nanoparticles can exhibit pseudocapacitance two ways: faradaic reactions in aqueous solvents (such as a solution of Na_2_SO_4_) and “extrinsic” pseudocapacitance through Li^+^ intercalation in organic electrolytes at sites located at the surface or near-surface regions^9^,^31^,^39^,^40^,^41^,^42^,^43^,^44^. When operating in the “extrinsic” pseudocapacitor mode, a nanoparticle MnO_x_ based device would be able to support rapid charge and discharge times due to the extremely short intercalation distances (5 nm or less), operating much more like a supercapacitor and with increased power densities. There has even been at least one report of MnO_2_ in an oxide supercapacitor application where a solvent containing multivalent (in this case Ca^2+^) ions was used^45^. The ability to use organic electrolytes provides a way to boost the electrochemical window of the device where necessary and boost both the operating voltage and energy density. Nevertheless, in some situations it can be beneficial to have a device that can operate using an aqueous electrolyte and avoid some of the safety and other concerns of operating with organic electrolytes. All of these different options and modes of operation makes a MnO_x_ based nanoparticle structure extremely versatile in the field of energy storage.

The tethered nanoparticle structure presented here addresses many of the current limitations in electrochemical storage devices. The need for rapid charge and discharge times, leading to enhanced power densities, and the ability to operate with larger voltage windows were partially addressed previously. Additional enhancements to charge transfer time would be seen from a tethered nanoparticle structure. It is no secret that almost all of the top battery and pseudocapacitor materials, as oxides, are extremely poor conductors^3^,^14^,^24^,^46^,^47^. The tethered particle architecture takes a step beyond the addition of conductive carbon by limiting the distance charge must travel through an oxide material and providing short conductive “wires” connected to a current collector to rapidly extract the charge after generation in the active material^1^,^9^,^11^. Eliminating the conductive carbon additives and binders by replacing them with conjugated organic linkers results in devices with significantly reduced inactive electrode material. In fact, when excluding the current collector, a tethered nanoparticle electrode has 99.9% active material by mass as opposed to roughly 70–80% active material (by mass) for more traditionally processed electrodes containing binder and conductive carbon. Nanoparticulate active material also address the limited cycle life in electrochemical electrodes, including those based on MnO_x_ materials^2^,^17^,^19^,^24^. As discrete and free standing nanoparticles, the active material should be well positioned to accommodate significant strain and size change as cations are (de)intercalated or surface chemistry changes occur. Furthermore, the tethered nanoparticle electrode discussed here is not only a step towards the rational design of oxide supercapacitor electrodes, Li battery electrodes, and Li-air catalysts, but also a platform to study behaviors such as charge de(insertion), conversion, and transport in a model system. Many parameters in the tethered nanoparticle electrode can be modified (active material chemistry, structure, size, and morphology; conductive linker chemistry; spacing of active material; etc.) and the effect of those modifications studied.

As discussed above, our choice of active material are the manganese oxides, which in addition to their broad range of potential applications (dependent on the choice of a particular MnO_x_ phase), are widely regarded as excellent charge storage materials due to the availability of multiple oxidation states and the structural ability to incorporate cations^32^,^33^,^34^,^45^. Furthermore, manganese oxides are very low in cost, particularly when compared to other materials often used in oxide supercapacitors (RuO_2_) and many Li-ion cathode materials, especially those rich in cobalt and nickel (LiCoO_2_ and Li(NiMnCo)O_2_). For example, although RuO_2_ has a very high specific capacitance (~1000 F/g), a RuO_2_ based capacitor large enough to power an electric vehicle would cost over $1 million^5^. On the other hand, manganese is the 12^th^ most abundant element found in the earth’s crust with Ti and Fe being the only transition metals found in higher quantities^48^.

In this work, we sought to demonstrate the viability of self-assembling an array of tethered nanoparticles, specifically MnO, as a robust synthesis for these nanoparticles had previously been reported. Modification of the surface chemistry of the particles to allow tethering to gold current collectors was a logical progression forward^49^. Optimization of the stoichiometry and phase of the manganese oxide would be the subject of future work. Additionally, the MnO nanoparticle synthesis conditions chosen allowed us to restrict the size of the nanoparticles to 20 nm or less (and therefore limit cation insertion distance to a maximum of 10 nm). Furthermore, the synthetic route chosen can also yield Mn_3_O_4_ nanoparticles of a similar size simply by excluding water as a reagent^49^. Finally, it has been reported that Mn_3_O_4_ particles can be converted to MnO_2_ via an *in situ* electrooxidation process^50^. Therefore, the single synthesis route reported here can easily produce an array of MnO, Mn_3_O_4_, or MnO_2_ nanoparticles tethered to a current collector.

## Results

### Synthesis and Characterization of Self-Assembled Electrodes

We searched the literature for a high yield and straightforward route to produce manganese oxide nanoparticles with a diameter on the order of 10 nm. Another criterion was to find a synthesis that used ligands which could easily be exchanged, allowing for further functionalization to form a linkage with a self-assembled monolayer (SAM). Several syntheses were identified but a few proved to be irreproducible in our laboratory. A communication published by Seo *et al*. provided a reproducible and straightforward route to synthesize MnO nanoparticles with oleylamine ligands and also allowed for size control by varying the reaction temperature^49^.

**Figure 1:**
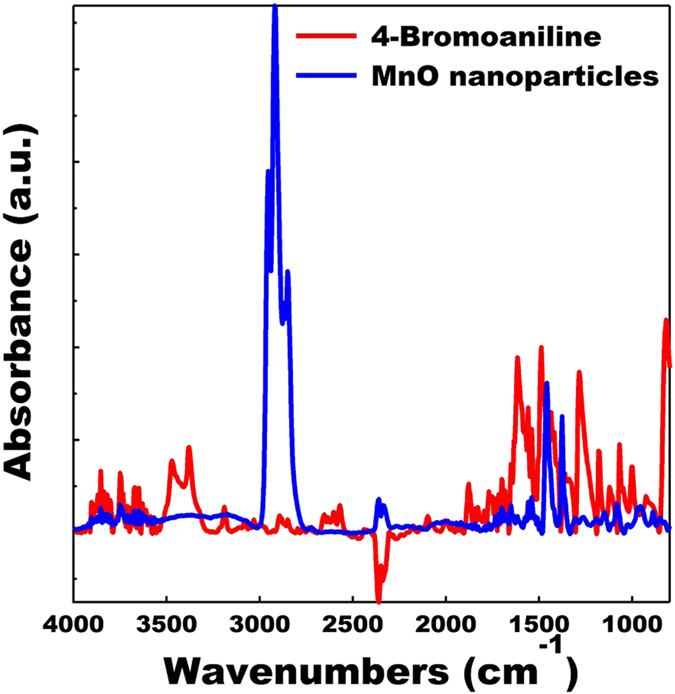
FTIR spectra of MnO nanoparticles synthesized with 4-bromoaniline ligands (blue). The FTIR spectra of neat 4-bromoaniline (red) is provided as a reference.

**Figure 2:**
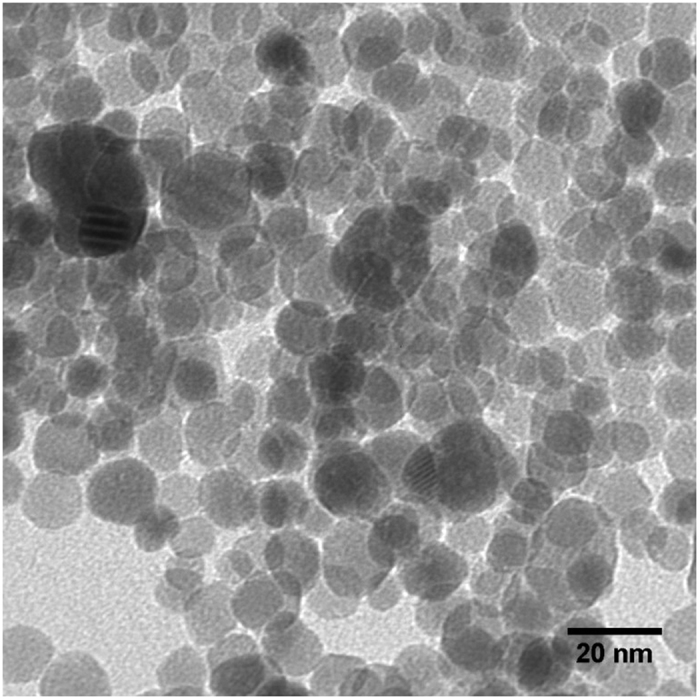
TEM image of MnO nanoparticles synthesized with 4-bromoaniline ligands.

We hypothesized that it should be feasible to replace the oleylamine ligands used in the synthesis published by Seo *et al*.^49^ with another amine terminated ligand containing a more reactive end group to form the SAM linkage. 4-bromoaniline was selected as the bromide moiety should allow for further functionalization. The conjugated structure of the phenyl group is electrically conducting and would facilitate fast charge transport from the tethered nanoparticles to the current collector. The synthesis of MnO nanoparticles with 4-bromoaniline exchanged for oleylamine proceeded as expected with only a few small changes, as outlined in the experimental section. Fourier transform infrared spectroscopy (FTIR) data of the MnO nanoparticles, synthesized with 4-bromoaniline are displayed in Fig. 1. The FTIR spectra of neat 4-bromoaniline is provided as a reference. In addition to a successful nanoparticle synthesis as confirmed via transmission electron microscopy (TEM), the FTIR spectra of the nanoparticles contained absorption peaks associated with the phenyl stretching mode (ν = 1458 cm^−1^) and CH stretching modes (ν = 2954 cm^−1^, 2920 cm^−1^, 2850 cm^−1^), which are strong indications that the 4-bromoaniline was bound to the surface of the MnO nanoparticles. Similar shifts to lower frequency have been reported upon aniline binding to other transition metals^51^,^52^. In addition to the FTIR spectra in Fig. 1, a TEM image of the functionalized MnO nanoparticles is displayed in Fig. 2. The TEM image confirms that essentially all of the synthesized MnO nanoparticles had a diameter under 20 nm.

**Figure 3:**
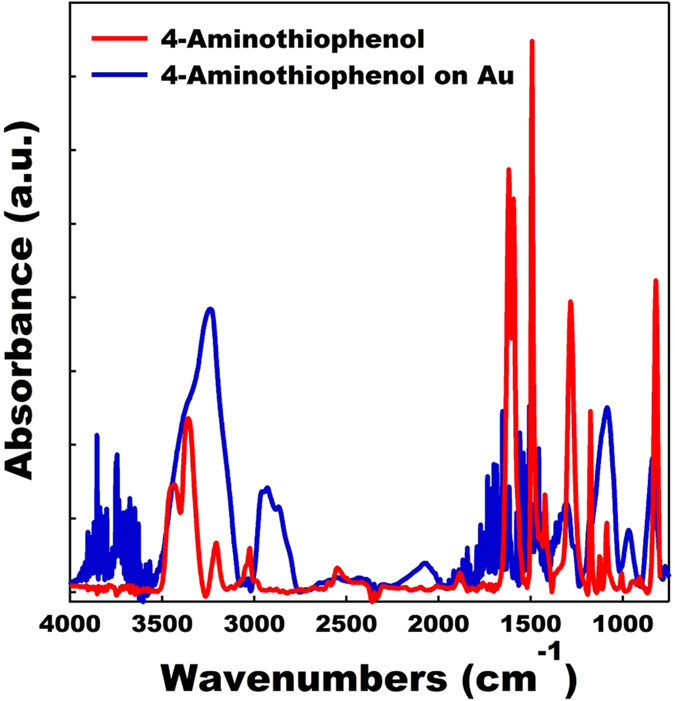
FTIR spectra of 4-aminothiophenol self-assembled monolayer deposited on a gold substrate (blue). The FTIR spectra of neat 4- aminothiophenol (red) is provided as a reference.

The next step in preparing a self-assembled array of tethered nanoparticles was to prepare a SAM on a suitable current collector. Gold was chosen as the current collector since thiol based SAMs on gold are extremely well characterized, their preparation straightforward, and several studies completed on the electron transport in thiol based molecular junctions^53^,^54^,^55^,^56^,^57^. Furthermore, there is an extensive array of commercially available thiol terminated molecules with many different end groups. The SAM would need to be terminated with an end group which could react with and form a bond with the bromine terminated 4-bromoaniline molecules serving as ligands on the nanoparticles. Thus, 4-aminothiophenol was chosen as it gave us a route to form a SAM on a gold current collector with an amine termination, which could react with the bromine end group on the MnO particles via an amination reaction. Once again, the conjugated structure of the phenyl group in 4-aminothiophenol is electron conducting and will facilitate fast charge transport from the tethered nanoparticles to the current collector^58^,^59^,^60^. The 4-aminothiophenol SAM was prepared using standard routes found in the literature and the growth of the SAM confirmed using FTIR spectroscopy (see Fig. 3)^56^,^57^. The FTIR spectra of neat 4-aminothiophenol is provided as a reference. Strong indications of the formation of a 4-aminothiophenol SAM include the presence of the following active IR modes: *ν*(NH_2_) = 3238 cm^−1^, *ν*(CH) = 2931 cm^−1^, *ν*_*s*_(SH) = 2549 cm^−1^, *ν*(CN) = 1085 cm^−1^, *γ*(CH) = 838 cm^−1^. The presence of the phenyl group stretching mode overlaps with water peaks present in the SAM spectra and due to the weaker absorption signal associated with attenuated total reflectance (ATR) FTIR measurements of the SAM they were not discernable.

**Figure 4:**
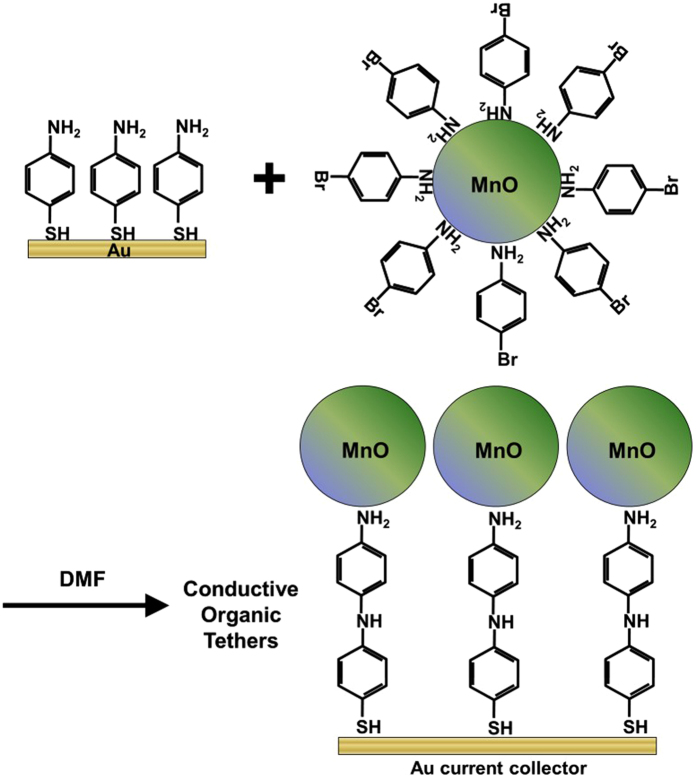
Schematic of the process for the reaction of a 4-aminothiophenol SAM on gold with 4-bromoaniline functionalized MnO particles to form an array of tethered nanoparticles. After formation of the tethered nanoparticle structure, a largely conductive pathway exists between the MnO nanoparticles and the gold current collector.

The final step in forming a tethered array of MnO nanoparticles was in making a chemical linkage between the nanoparticle ligands and the self-assembled monolayer (SAM). An amination reaction between the bromine termination on the 4-bromoaniline and the 4-aminothiophenol forms a linkage between the particles and the SAM. Figure 4 shows a schematic of how the SAM on gold and functionalized MnO particles react and form an array of tethered nanoparticles. When the reaction is complete, a largely conjugated pathway exists between the MnO nanoparticles and the gold current collector. Charge tunneling would still need to occur over very short (~ 2 Å) distances at the particle surface, the secondary amine linkage, and at the interface between the thiol group and the gold surface, although evidence exists showing sufficient electron transport should still occur^58^,^61^.

**Figure 5:**
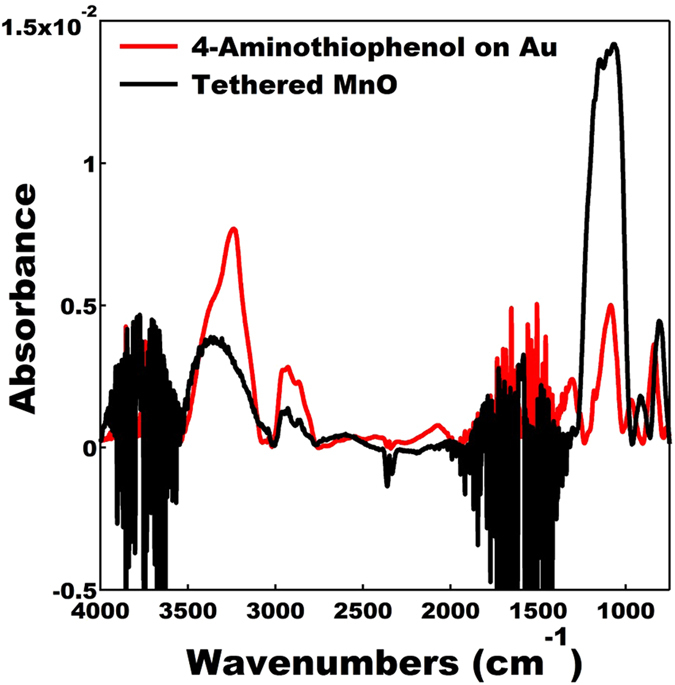
FTIR spectra of an array of MnO nanoparticles bound to a gold substrate via a short conductive organic linkage (black). The FTIR spectra of a 4-aminothiophenol self-assembled monolayer deposited on a gold substrate (red) is provided as a reference.

We deliberately avoided the use of catalysts to aid the amination reaction in order to exclude impurities. Nevertheless, it is certainly possible that Pd, Cu, or CsOH based catalysts could make the amination reaction more efficient. We allowed the nanoparticles and SAM on gold to react in a solution of dimethylformamide (DMF) over a period of 48 hrs. We did experiment with elevated temperatures (50 °C) to make the reaction more efficient but there was evidence that either decomposition of the organic ligands, decomposition of the SAM, and/or undesirable side reactions were occurring. A FTIR spectra of the tethered MnO nanoparticles, with the spectra of the 4-aminothiophenol SAM on gold provided as a reference, is displayed in Fig. 5. Strong indication of the formation of the linkage between the nanoparticles and the gold substrate is evident in the following active IR modes: *ν*(NH_2_) = 3359 cm^−1^, *ν*(CH) = 2929 cm^−1^, *ν*(CN) = 1070 cm^−1^, *γ*(CH) = 806 cm^−1^. The increase in the area under the *ν*(CN) peak gives us additional confidence that a secondary amine linkage was formed and the number of CN bonds for the system doubled.

**Figure 6:**
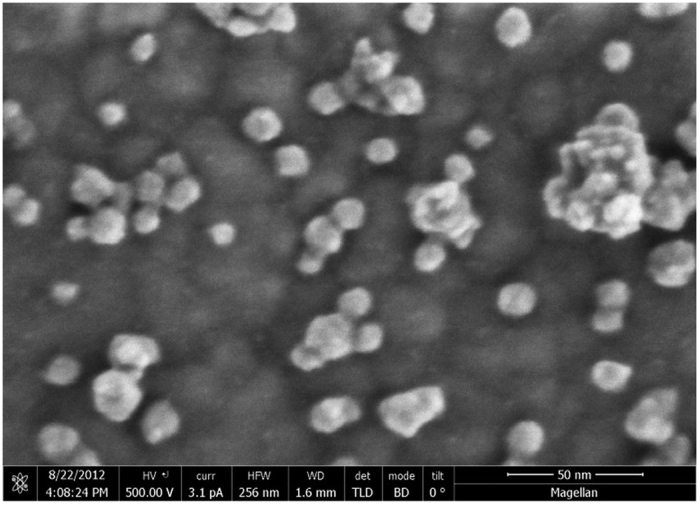
High resolution SEM of array of MnO nanoparticles tethered to a gold surface.

Further characterization of the self-assembled array of nanoparticles (and confirmation of a successful synthesis) was provided through high resolution scanning electron microcrope (SEM) imaging of the nanoparticle SAM. A high resolution SEM image of the surface is shown in Fig. 6. In the SEM image, individual particles bound to the gold surface can be resolved. In some regions, the particles aggregated together into small clumps on the gold surface consisting of roughly 3–15 particles. This is not too surprising given the high surface area of the nanoparticles and short length (~4 Å) of their ligands, which would not provide significant steric hindrance to prevent particle agglomeration either in solution or on the gold surface. Clustering of individual particles can also be observed in TEM images (see Fig. 2). Particle stabilization and separation both in solution and on the gold surface can be further optimized through careful choice of ligand molecules with similar functionality and increased chain length. However, the choice of ligand length would need to be balanced with the need for good charge transfer.

## Discussion

A self-assembled array of MnO nanoparticles bound directly to a gold current collector using short (~1.5 nm) conducting organic linkers was synthesized and characterized. This was accomplished by incorporating complementary reactive organic ligands to both MnO nanoparticles and a gold substrate. Dimethylformamide (DMF) proved to be the most effective solvent for this reaction with results confirmed by FTIR spectroscopy. This array of tethered nanoparticles, and new arrays fabricated in a similar fashion, can have an immediate impact as a new route to fabricate oxide supercapacitor electrodes, Li battery electrodes, and Li-air battery catalysts that can operate in both aqueous and organic electrolytes. Furthermore, tethered nanoparticles are a step towards addressing some of the most difficult challenges facing electrochemical energy storage: achieving high energy and power density in the same device, increasing cycle life through a structure more impervious to degradation, decreasing inactive electrode material, and improving charge transfer.

The self-assembly synthesis approach presented here is amenable to coating both small and large scale areas up to and beyond a meter. Furthermore, this single synthetic route can easily produce an array of MnO, Mn_3_O_4_, or MnO_2_ nanoparticles tethered to a current collector through facile modification of the nanoparticle synthesis or by post synthesis electrochemical oxidation of the manganese. Additionally, the approach can be modified to accommodate a wide range of metal and metal oxide nanoparticles, organic linking chemistries, and substrates. As a logical next step, the electrochemical response of our tethered MnO nanoparticle array in both aqueous and organic electrolytes will be characterized. We also hope this work will excite other researchers to build upon these results and begin to address key problems in electrochemical energy storage.

## Methods

### Synthesis of MnO nanoparticles and self-assembled electrode

All chemicals and reagents were purchased from Sigma-Aldrich and used as received. Water used in the synthesis of the MnO nanoparticles was treated by a building wide DI water system and had a measured resistivity of 18.2 MΩ. The MnO nanoparticle synthesis followed the approach as described in the publication by Seo *et al*. with a few notable differences. Specifically, the oleylamine surfactant and solvent was replaced with 4-bromoaniline. A manganese(II) acetylacetonate or [Mn(acac)_2_]:4-bromoaniline:water molar ratio of 1:24:10 (0.9 g Mn(acac), 14.6 g 4-bromoaniline, and 0.630 g water) was used and the synthesis was run at 200 °C for 12 hours. The resulting brown suspension was cooled to room temperature and the particles recovered by centrifugation for 15 min. at 4500 rpm and the supernatant removed. A mixture of 10 mL of ethanol and 20 mL of hexanes was added to the brown precipitate, which was vortex mixed and sonicated for 15 min. at 50 °C to redisperse the particles. The particles were pelleted by centrifuging at 4500 rpm for 15 min. and resuspended in hexanes.

4-aminothiophenol monolayers were formed on 1 cm square gold coated silicon wafers (SPI Supplies; West Chester, PA). The substrates were sonicated in ethanol for 20 min. and then cleaned using UV/ozone for an additional 20 minutes just prior to immersion in a 1 mmol solution of 4-aminothiophenol in ethanol for a period of 48 hrs. At the completion of the 48 hr. soak, the SAM coated substrates were removed from the 4-aminothiophenol solution, rinsed with ethanol, and blown dry with nitrogen. In order to form the layer of tethered MnO nanoparticles, a 100 μL solution of MnO particles dispersed in hexanes were added to 10 mL of dimethylformamide (DMF) and allowed to soak at room temperature for a period of 48 hr. along with the SAM coated gold substrate. After the 48 hr. soak time, the nanoparticle coated substrates were removed from the solution, rinsed with ethanol, and blown dry with nitrogen.

### FTIR spectroscopy

Fourier Transform – infrared (FTIR) spectra were obtained with a Bruker IFS 66 S FTIR spectrometer (Bruker Optics Inc.; Billerica, MA) in a sample chamber purged with nitrogen gas. MnO nanoparticles were pressed into a KBr pellet with a sample concentration of 2% (w/w). Reference spectra of neat 4-aminothiophenol and 4-bromoaniline were collected by depositing the liquid samples between two KBr windows. FTIR spectra of the 4-aminothiophenol monolayers and tethered MnO nanoparticles were collected via the reflectance-absorbance FTIR spectroscopy (RAIRS) technique using the Seagull accessory from Harrick Scientific (Pleasantville, NY).

### Electron microscopy and analysis

Bright field TEM images were acquired with a JEOL 1200 EX (Tokyo, Japan) using an acceleration voltage of 120 kV. The instrument has a point to point resolution of approximately 9 Å. Images were collected on an Advanced Microscopy Techniques (AMT) XR280L-A 2.8 Mpixel CMOS camera (AMT; Woburn, MA). Samples for TEM analysis were prepared by depositing the nanoparticles onto a holey carbon coated copper TEM grid (Structure Probe, Inc.; West Chester, PA). A 3 μL aliquot of MnO nanoparticles suspended in hexanes was withdrawn from a vial immediately after vigorously vortexing and then added to the surface of the TEM grid. Filter paper placed under the TEM grid was used to aid in wicking away excess solvent and to evenly disperse the nanoparticles across the holey carbon surface.

SEM images were collected using the FEI Magellan 400 Extreme High Resolution (XHR) SEM. Images displayed in the publication were taken at a 1.6 mm Working Distance with a 500 V/3.1 pA beam in Beam Deceleration (BD) mode after an *in situ* 2 minute plasma clean of the sample. In this BD mode, a primary beam energy of 1 kV was emitted from the Field Emitter gun and combined with a beam deceleration of 500 V at the sample, which yielded a 500 V beam on the sample surface. This low energy beam voltage provided high resolution imaging with high surface sensitivity, minimizing sample interference without sacrificing resolution and contrast.

All plots were generated using Igor Pro software (WaveMetrics, Inc.; Lake Oswego, OR, USA).

## Additional Information

**How to cite this article**: Stevens, T. E. *et al*. Self-Assembled Array of Tethered Manganese Oxide Nanoparticles for the Next Generation of Energy Storage. *Sci. Rep.* **7**, 44191; doi: 10.1038/srep44191 (2017).

**Publisher's note:** Springer Nature remains neutral with regard to jurisdictional claims in published maps and institutional affiliations.

## References

[b1] Tarascon J. M. Key challenges in future Li-battery research. Philosophical Transactions of the Royal Society A: Mathematical, Physical and Engineering Sciences 368, 3227–3241 (2010). 10.1098/rsta.2010.0112 20566508

[b2] in Report of the Basic Energy Sciences Workshop on Electrical Energy Storage (Office of Basic Energy Sciences, Department of Energy, 2007).

[b3] Simon P. & Gogotsi Y. Materials for electrochemical capacitors. Nat Mater 7, 845–854 (2008). 18956000 10.1038/nmat2297

[b4] Whittingham M. S. Materials Challenges Facing Electrical Energy Storage. MRS Bull. 33, 411–419 (2008).

[b5] Miller J. R. & Simon P. Electrochemical Capacitors for Energy Management. Science 321, 651–652 (2008). 18669852 10.1126/science.1158736

[b6] Abruna H. D., Kiya Y. & Henderson J. C. Batteries and electrochemical capacitors. Physics Today 61, 43–47 (2008).

[b7] Patil A. . Issue and challenges facing rechargeable thin film lithium batteries. Mater. Res. Bull. 43, 1913–1942 (2008).

[b8] Min M., Machida K., Jang J. H. & Naoi K. Hydrous RuO[sub 2]/Carbon Black Nanocomposites with 3D Porous Structure by Novel Incipient Wetness Method for Supercapacitors. J. Electrochem. Soc. 153, A334 (2006).

[b9] Arico A. S., Bruce P., Scrosati B., Tarascon J.-M. & van Schalkwijk W. Nanostructured materials for advanced energy conversion and storage devices. Nat Mater 4, 366–377 (2005). 15867920 10.1038/nmat1368

[b10] Amatucci G. G., Badway F., Du Pasquier A. & Zheng T. An asymmetric hybrid nonaqueous energy storage cell. J. Electrochem. Soc. 148, A930–A939 (2001).

[b11] Armand M. & Tarascon J. M. Building better batteries. Nature 451, 652–657 (2008). 18256660 10.1038/451652a

[b12] Muldoon J. . Electrolyte roadblocks to a magnesium rechargeable battery. Energy & Environmental Science 5, 5941–5950 (2012).

[b13] Wang W. . A new cathode material for super-valent battery based on aluminium ion intercalation and deintercalation. Sci. Rep. 3 (2013). 10.1038/srep03383 PMC3843166 24287676

[b14] Zhang H., Yu X. & Braun P. V. Three-dimensional bicontinuous ultrafast-charge and -discharge bulk battery electrodes. Nat Nano 6, 277–281 (2011). 10.1038/nnano.2011.38 21423184

[b15] Belanger D., Brousse T. & Long J. W. Manganese oxides: battery materials make the leap to electrochemical capacitors. Electrochem. Soc. Interface 17, 49–52 (2008).

[b16] Miller J. R. & Burke A. F. Electrochemical capacitors: challenges and opportunities for real-world applications. Electrochem. Soc. Interface 17, 53–57 (2008).

[b17] Luo W., Hu X., Sun Y. & Huang Y. Controlled Synthesis of Mesoporous MnO/C Networks by Microwave Irradiation and Their Enhanced Lithium-Storage Properties. ACS Applied Materials & Interfaces 5, 1997–2003 (2013). 23432367 10.1021/am302813d

[b18] Fang X. . Electrode reactions of manganese oxides for secondary lithium batteries. Electrochem. Commun. 12, 1520–1523 (2010).

[b19] Liu B. . Encapsulation of MnO Nanocrystals in Electrospun Carbon Nanofibers as High-Performance Anode Materials for Lithium-Ion Batteries. Sci. Rep. 4 (2014). 10.1038/srep04229 PMC3944319 24598639

[b20] Li X. . Interconnected porous MnO nanoflakes for high-performance lithium ion battery anodes. J. Mater. Chem. 22, 9189–9194 (2012).

[b21] Zhong K. . Investigation on porous MnO microsphere anode for lithium ion batteries. J. Power Sources 196, 6802–6808 (2011).

[b22] Wang T., Peng Z., Wang Y., Tang J. & Zheng G. MnO Nanoparticle@Mesoporous Carbon Composites Grown on Conducting Substrates Featuring High-performance Lithium-ion Battery, Supercapacitor and Sensor. Sci. Rep. 3 (2013). 10.1038/srep02693 PMC3776197 24045767

[b23] Zhong K. . MnO powder as anode active materials for lithium ion batteries. J. Power Sources 195, 3300–3308 (2010).

[b24] Sun B., Chen Z., Kim H.-S., Ahn H. & Wang G. MnO/C core–shell nanorods as high capacity anode materials for lithium-ion batteries. J. Power Sources 196, 3346–3349 (2011).

[b25] Liu S.-Y. . Nanocrystal manganese oxide (Mn3O4, MnO) anchored on graphite nanosheet with improved electrochemical Li-storage properties. Electrochim. Acta 66, 271–278 (2012).

[b26] Yu X. Q. . Nanocrystalline MnO thin film anode for lithium ion batteries with low overpotential. Electrochem. Commun. 11, 791–794 (2009).

[b27] Si W. . On chip, all solid-state and flexible micro-supercapacitors with high performance based on MnOx/Au multilayers. Energy & Environmental Science 6, 3218–3223 (2013).

[b28] Kim J.-H., Lee K. H., Overzet L. J. & Lee G. S. Synthesis and Electrochemical Properties of Spin-Capable Carbon Nanotube Sheet/MnOx Composites for High-Performance Energy Storage Devices. Nano Lett. 11, 2611–2617 (2011). 21661756 10.1021/nl200513a

[b29] Wu M.-S., Chiang P.-C. J., Lee J.-T. & Lin J.-C. Synthesis of Manganese Oxide Electrodes with Interconnected Nanowire Structure as an Anode Material for Rechargeable Lithium Ion Batteries. The Journal of Physical Chemistry B 109, 23279–23284 (2005). 16375294 10.1021/jp054740b

[b30] Johnson C. S. Development and utility of manganese oxides as cathodes in lithium batteries. J. Power Sources 165, 559–565 (2007).

[b31] Xu C., Kang F., Li B. & Du H. Recent progress on manganese dioxide based supercapacitors. J. Mater. Res. 25, 1421–1432 (2010).

[b32] Wei W., Cui X., Chen W. & Ivey D. G. Manganese oxide-based materials as electrochemical supercapacitor electrodes. Chem. Soc. Rev. 40, 1697–1721 (2011). 21173973 10.1039/c0cs00127a

[b33] Thackeray M. M. Manganese oxides for lithium batteries. Prog. Solid State Chem. 25, 1–71 (1997).

[b34] Thackeray M. M. . The versatility of MnO2 for lithium battery applications. J. Power Sources 43, 289–300 (1993).

[b35] Christensen J. . A Critical Review of Li/Air Batteries. J. Electrochem. Soc. 159, R1–R30 (2012).

[b36] Debart A., Paterson A. J., Bao J. & Bruce P. G. alpha-MnO(2) nanowires: A catalyst for the O(2) electrode in rechargeable lithium batteries. Angewandte Chemie-International Edition 47, 4521–4524 (2008). 18461594 10.1002/anie.200705648

[b37] Girishkumar G., McCloskey B., Luntz A. C., Swanson S. & Wilcke W. Lithium - Air Battery: Promise and Challenges. Journal of Physical Chemistry Letters 1, 2193–2203 (2010).

[b38] Lu Y.-C., Gasteiger H. A., Crumlin E., McGuire R. Jr. & Shao-Horn Y. Electrocatalytic Activity Studies of Select Metal Surfaces and Implications in Li-Air Batteries. J. Electrochem. Soc. 157, A1016–A1025 (2010).

[b39] Simon P., Gogotsi Y. & Dunn B. Where Do Batteries End and Supercapacitors Begin? Science 343, 1210–1211 (2014). 24626920 10.1126/science.1249625

[b40] Conway B. E. Transition from “Supercapacitor” to “Battery” Behavior in Electrochemical Energy Storage. J. Electrochem. Soc. 138, 1539–1548 (1991).

[b41] Conway B. E. Electrochemical Supercapacitors: Scientific Fundamentals and Technological Applications. (Springer, 1999).

[b42] Augustyn V. . High-rate electrochemical energy storage through Li+ intercalation pseudocapacitance. Nat Mater 12, 518–522 (2013). 23584143 10.1038/nmat3601

[b43] Okubo M. . Nanosize Effect on High-Rate Li-Ion Intercalation in LiCoO2 Electrode. J. Am. Chem. Soc. 129, 7444–7452 (2007). 17511453 10.1021/ja0681927

[b44] Xu C., Li B., Du H., Kang F. & Zeng Y. Supercapacitive studies on amorphous MnO2 in mild solutions. J. Power Sources 184, 691–694 (2008).

[b45] Xu C., Du H., Li B., Kang F. & Zeng Y. Capacitive Behavior and Charge Storage Mechanism of Manganese Dioxide in Aqueous Solution Containing Bivalent Cations. J. Electrochem. Soc. 156, A73–A78 (2009).

[b46] Chung S.-Y., Bloking J. T. & Chiang Y.-M. Electronically conductive phospho-olivines as lithium storage electrodes. Nat Mater 1, 123–128 (2002). 12618828 10.1038/nmat732

[b47] Lu Q., Chen J. G. & Xiao J. Q. Nanostructured Electrodes for High-Performance Pseudocapacitors. Angew. Chem. Int. Ed. 52, 1882–1889 (2013). 10.1002/anie.201203201 23307657

[b48] Greenwood N. N. & Earnshaw A. Chemistry of the Elements, first ed. (Pergamon Press, 1984).

[b49] Seo W. S. . Size-dependent magnetic properties of colloidal Mn3O4 and MnO nanoparticles. Angewandte Chemie-International Edition 43, 1115–1117 (2004). 14983449 10.1002/anie.200352400

[b50] Xiao W., Xia H., Fuh J.-Y.-H. & Lu L. Electrochemical Synthesis and Supercapacitive Properties of ε-MnO2 with Porous/Nanoflaky Hierarchical Architectures. J. Electrochem. Soc. 156, A627–A633 (2009).

[b51] Lee-Thorp J. A., Rüede J. E. & Thornton D. A. The infrared spectra (3500—150 cm-1) of aniline complexes of cobalt(II), nickel(II), copper(II) and zinc(II) halides. Journal of Molecular Structure 50, 65–71 (1978).

[b52] Raman N., Ravichandran S. & Thangaraja C. Copper(II), cobalt(II), nickel(II) and zinc(II) complexes of Schiff base derived from benzil-2,4-dinitrophenylhydrazone with aniline. Journal of Chemical Sciences 116, 215–219 (2004).

[b53] Tao N. J. Electron transport in molecular junctions. Nat Nano 1, 173–181 (2006). 10.1038/nnano.2006.130 18654182

[b54] Holmlin R. E. . Electron Transport through Thin Organic Films in Metal−Insulator−Metal Junctions Based on Self-Assembled Monolayers. J. Am. Chem. Soc. 123, 5075–5085 (2001). 11457338 10.1021/ja004055c

[b55] Zhou Y. . First-principles study of length dependence of conductance in alkanedithiols. The Journal of chemical physics 128, 044704–044704 (2008). 18247978 10.1063/1.2827868

[b56] Bain C. D. . Formation of monolayer films by the spontaneous assembly of organic thiols from solution onto gold. J. Am. Chem. Soc. 111, 321–335 (1989).

[b57] Allara D. L. & Nuzzo R. G. Spontaneously organized molecular assemblies. 2. Quantitative infrared spectroscopic determination of equilibrium structures of solution-adsorbed n-alkanoic acids on an oxidized aluminum surface. Langmuir 1, 52–66 (1985).

[b58] Samanta M. P., Tian W., Datta S., Henderson J. I. & Kubiak C. P. Electronic conduction through organic molecules. Physical Review B 53, R7626–R7629 (1996). 10.1103/physrevb.53.r7626 9982283

[b59] Heeger A. J. Semiconducting and Metallic Polymers: The Fourth Generation of Polymeric Materials. The Journal of Physical Chemistry B 105, 8475–8491 (2001). 10.1002/1521-3773(20010716)40:14<2591::AID-ANIE2591>3.0.CO;2-0 29712324

[b60] Peet J. . Efficiency enhancement in low-bandgap polymer solar cells by processing with alkane dithiols. Nat Mater 6, 497–500 (2007). 17529968 10.1038/nmat1928

[b61] Wessels J. M. . Optical and Electrical Properties of Three-Dimensional Interlinked Gold Nanoparticle Assemblies. Journal of the American Chemical Society 126, 3349–3356 (2004). 15012165 10.1021/ja0377605

